# West Nile Virus in California

**DOI:** 10.3201/eid1008.040077

**Published:** 2004-08

**Authors:** William Reisen, Hugh Lothrop, Robert Chiles, Minoo Madon, Cynthia Cossen, Leslie Woods, Stan Husted, Vicki Kramer, John Edman

**Affiliations:** * University of California, Davis, California, USA;; † Greater Los Angeles County Mosquito and Vector Control District, Santa Fe Springs, California, USA;; ‡ California Department of Health Services, Sacramento, California, USA; 1 Procedures for the bleeding and husbandry of sentinel chickens were described in Protocol 9608 approved by the University of California, Davis, Animal Use and Care Administrative Advisory Committee.; 2 The collection, banding, and bleeding of wild birds were conducted under Protocol 9605 approved by the Animal Use and Care Administrative Advisory Committee of the University of California, Davis, California Resident Scientific Collection Permit 801049-02 by the State of California Department of Fish and Game, and Master Station Federal Bird Marking and Salvage Permit 22763 from the U.S. Geological Survey Bird Banding Laboratory.

**Keywords:** West Nile virus, California, St. Louis encephalitis virus, surveillance, invasion, Culex tarsalis, Culex pipiens quinquefasciatus

## Abstract

West Nile virus (WNV) was first isolated in California during July 2003 from a pool of *Culex tarsalis* collected near El Centro, Imperial County. WNV transmission then increased and spread in Imperial and Coachella Valleys, where it was tracked by isolation from pools of *Cx. tarsalis*, seroconversions in sentinel chickens, and seroprevalence in free-ranging birds. WNV then dispersed to the city of Riverside, Riverside County, and to the Whittier Dam area of Los Angeles County, where it was detected in dead birds and pools of *Cx. pipiens quinquefasciatus*. By October, WNV was detected in dead birds collected from riparian corridors in Los Angeles, west to Long Beach, and through inland valleys south from Riverside to San Diego County. WNV was reported concurrently from Arizona in mid-August and from Baja, Mexico, in mid-November. Possible mechanisms for virus introduction, amplification, and dispersal are discussed.

## Abstract 2

The spread of WNV in California is tracked.

Since the arrival of West Nile virus (WNV, *Flavivirus*, *Flaviviridae*) into New York City in 1999, the public health community has chronicled the unimpaired spread of this virus across North America from the Atlantic to the Pacific Coasts (1) and from Canada (2) into tropical America (3) and the Caribbean (4,5). Regionally, the epidemic has been characterized by an initial introduction with a few human cases during the first season, followed by explosive amplification and an epidemic during the second season, and then subsidence to maintenance levels. Ongoing or recent transmission of closely related St. Louis encephalitis virus (SLEV) in Florida, Louisiana, and Texas seems to have had little dampening effect on WNV amplification, which contradicts the long-held premise that two closely related flaviviruses cannot co-exist (6). Minimal ecologic resistance or selection pressure has left the strains of WNV intact genetically (7,8), until relatively minor changes may have resulted in attenuation in Mexico (3).

In 1999, when WNV was introduced into North America, few encephalitis virus surveillance programs remained intact, and most were structured to protect urban centers (9). Consequently, the initial detection of WNV in most areas occurred after introduction and amplification and frequently was heralded by the discovery of dead crows or horses and humans with neurologic illness. California is somewhat unique in that an extensive arbovirus surveillance program has remained intact statewide. Because of endemic SLEV and western equine encephalomyelitis virus (WEEV, *Alphavirus*, *Togaviridae*) transmission and nuisance mosquito problems, California residents have supported special local mosquito and vector control districts that currently protect ca. 33.9 million people (88% of the state's population) over a combined area of ca. 166,107 km^2^. The associated California Encephalitis Virus Surveillance Program, which has been in place for more than 35 years (10), monitors mosquito abundance and infection rates as well as virus transmission to sentinel chickens. Local surveillance programs are coordinated at the state level by the California Department of Health Services, and supporting diagnostics currently are conducted by that agency and the Center for Vectorborne Diseases at the University of California, Davis. Recent ecologic studies on virus persistence and amplification by that center have been set against this extensive surveillance backdrop and have focused on wetlands along the Salton Sea (11,12). Our current study describes how this surveillance program, extended by associated field research projects, provided an early warning of the arrival of WNV in California and preliminary information on its ecology, surveillance, and dispersal during 2003. Highlighted information includes climatic conditions, the possible route (s) of introduction and subsequent dispersal, abundance of vector populations at the time of invasion, avian populations involved, and the comparative sensitivity of different surveillance indicators in different ecologic settings.

## Materials and Methods

Climate data from Coachella Valley and the Los Angeles basin were downloaded from National Oceanographic and Atmospheric Administration weather stations from the California Integrated Pest Management website (http://www.ipm.ucdavis.edu/). These data were included to describe temperature conditions when virus was active and rainfall events associated with the intrusion of moist monsoon conditions from the Gulf of Mexico.

**Figure 1:**
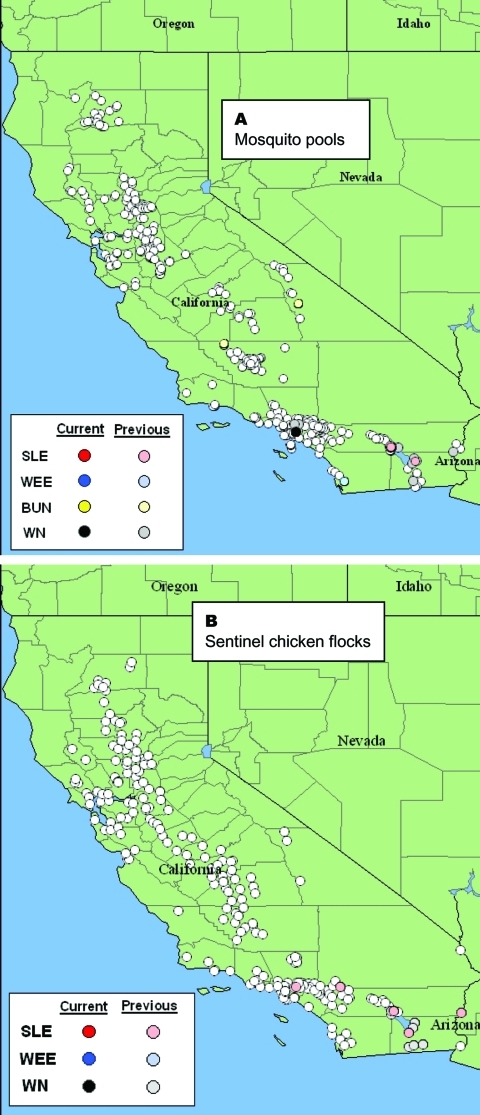
Map of California showing locations where A) 9,731 mosquito pools were collected and B) 212 sentinel chicken flocks were located through November 1, 2003. Data are cumulative for 2003 and show negative, previously positive, and currently active sites as downloaded from http://www.vector.ucdavis.edu/. SLE, St. Louis encephalitis virus; WEE, western equine encephalitis virus; BUN, viruses in the California encephalitis virus complex, family *Bunyaviridae*; WN, West Nile virus.

Mosquitoes were collected biweekly at permanent sites by using dry ice-baited CDC-style traps (CO_2_ traps) operated without light (13) and gravid female traps (14). Sampling effort varied spatially. Six and 42 CO_2_ traps were operated at wetlands and agricultural habitats in Imperial and Coachella Valleys, respectively, whereas 4–13 CO_2_ and 6–20 gravid traps were operated per sampling occasion within an 8-km radius of the Whittier Dam area of Los Angeles. Mosquitoes were anesthetized with triethylamine, enumerated by species, grouped into pools of <50 females per species per site, frozen at –80°C, and then shipped on dry ice to the University of California at Davis for testing. There, mosquitoes were screened for infectious virus by cell culture by using an in situ enzyme immunoassay (EIA) (15) and for viral RNA using a robotic TaqMan system (16). Three separate TaqMan assays were conducted on each pool to detect WNV, SLEV, and WEEV by using primer sets evaluated previously against historical California lineages of SLEV and WEEV (E.N. Green and W.K. Reisen, unpub. data). Locations of mosquito pool collection sites statewide during 2003 are shown in Figure 1A.

Statewide, 212 sentinel flocks of 10 white leghorn hens each^1^ were bled biweekly by lancet prick of the comb and samples mailed to the Viral and Rickettsial Diseases Laboratory, California Department of Health Services, where they were screened for antibody by WEEV or WNV/SLEV antigens with an EIA (17). *Flavivirus*-positive hens were re-bled, and whole serum specimens were tested by endpoint plaque reduction neutralization tests (PRNT) to separate those with antibody to WNV or SLEV. The locations of sentinel chicken flocks sampled during the summer of 2003 are summarized in Figure 1B. Three and six flocks, respectively, were located at research areas in Imperial and Coachella Valleys near the Salton Sea, whereas a single flock was located in the Whittier area of Los Angeles County. Seropositive birds were replaced at these study sites to track virus transmission activity through the season.

Free-ranging birds^2^ were collected weekly at two wetland sites along the north shore of the Salton Sea by using 8 to 10 mist nets and 1 to 2 grain-baited ground traps, as described previously (18). Additional grain-baited traps were deployed at seven sites throughout Coachella Valley. Birds were identified to species, sex, and age; leg-banded with U.S. Geological Survey tags; bled by jugular puncture (0.1 mL whole blood into 0.9 mL of saline); and released. Samples were clarified by centrifugation and then screened for WEEV, SLEV, or WNV antibodies by using an EIA (19). Positive samples were retested by PRNT. Separation of SLEV and WNV infection was based on a fourfold or greater difference in endpoint PRNT titers.

Dead birds were reported to the California Department of Health Services by telephone. Carcasses appearing to be <24 hours old were submitted by local mosquito and vector control districts and public health agencies for necropsy to the California Animal Health and Food Safety laboratory at the University of California, Davis, where kidney, lung, and brain tissues were removed for testing. Kidney samples were screened for WNV RNA by using the robotic TaqMan system and primers described above. Virus isolation was attempted from pooled organs of RNA-positive birds by using a plaque assay on Vero cell culture.

## Results

**Figure 2:**
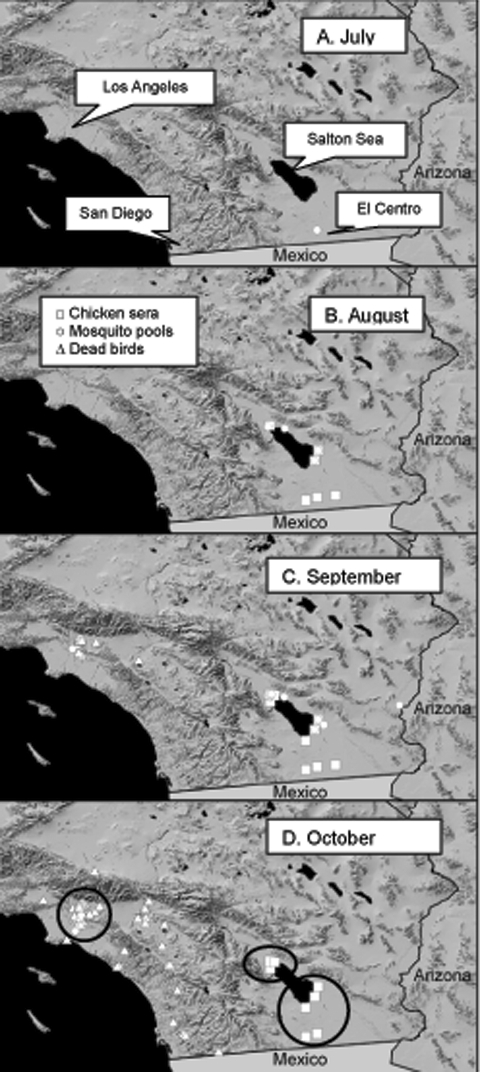
Introduction of West Nile virus into California. Panels show the locations of positive mosquito pools, sentinel chicken flocks with >1 seroconversion, and positive dead birds during each month. Encircled in panel D are the locations of the three foci studied in depth during 2003.

**Figure 3:**
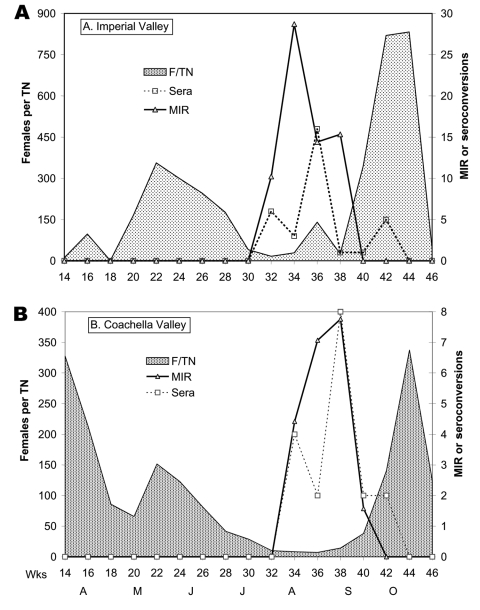
Virus temporal dynamics in relation to *Culex tarsalis* in A) Imperial and B) Coachella Valleys. Shown are female (F) *Cx. tarsalis* collected per CO_2_ trap night (TN). West Nile virus minimum infection rates (MIR) per 1,000 tested adjusted for differential sample sizes, and the number of sentinel chicken seroconversions per 2-week period.

WNV was probably introduced into California during July 2003 and was detected initially in a pool of *Cx. tarsalis* mosquitoes collected near El Centro, Imperial County, on July 16, 2003 (Figure 2). During the following weeks, WNV was isolated from 16 pools of *Cx. tarsalis*, and transmission was detected by 51 seroconversions of sentinel chickens at six flocks positioned on wildlife refuges along the southern shore of the Salton Sea and in agricultural habitats near the Mexican border (Figure 2). WNV was detected concurrently along the Colorado River at Yuma and in eastern Arizona by the Arizona surveillance system (20). Multiple isolations of SLEV were made in Arizona before WNV was first detected in August. WNV was not reported from Baja, Mexico, until November 2003 (21). At the time of WNV amplification in Imperial County, mosquito catches in CO_2_ traps along the southern shore of the Salton Sea had reached the typical midsummer minimum (Figure 3A) and were dominated by *Cx. tarsalis*, *Cx. erythrothorax*, and *Aedes vexans*. However, only pools of *Cx. tarsalis* contained WNV (Table 1). A comparable scenario developed in the Coachella Valley during mid-August (Figure 3B), with 10 isolations of WNV and 3 of SLEV made from *Cx. tarsalis* (even though 466 pools of other mosquito species were tested) and 20 seroconversions of sentinel chickens to both viruses detected at multiple flocks (Table 1). Despite intensive surveillance throughout the rest of Coachella Valley, WNV and SLEV activity was detected only along the north shore of the Salton Sea, even after the flooding of wetlands for migratory waterfowl in September resulted in a marked increase in *Cx. tarsalis* abundance (Figure 3A,B). No positive dead bird, human, or equine cases were associated with the initial invasion and amplification of WNV in rural southeast California, until a single human case was reported near El Centro in late October.

**Table 1:** Surveillance data for southern West Nile virus foci and the rest of California, January 1–November 1, 2003^a^

Surveillance data	Research areas				
	Imperial	Coachella	LA	Remaining agencies	Total
Human cases	1	0	0	1	2
Horse cases	0	0	0	1	1
Mosquito pools	238	1,414	1,663	6,416	9,731
*Culex tarsalis*	150	948	121	3,176	4,395
WNV pos	16	10	0	0	26
SLEV pos	1	3	0	0	4
WEEV pos	0	0	0	1	1
*Cx. pipiens complex*	0	299	1,036	1,170	2,505
WNV pos	0	0	6	0	6
Others^b^	88	167	506	2,070	2,831
Sentinel chickens	6	10	5	191	212
WNV pos	51	18	0	0	69
SLEV pos	3	2	0	8	13
WEEV pos	0	0	0	0	0
Dead birds reported	23	15	1,218	6,294	7,550
Tested	6	5	256	1,118	1,385
WNV pos	0	0	38	21	59
Wild bird sera	0	3,178	1,452	4,502	9,132
WNV pos		51	0	0	51
WEEV pos		2	0	0	2

^a^LA, Los Angeles; WNV, West Nile virus; SLEV, St. Louis encephalitis virus; WEEV, western equine encephalitis virus; pos, positive. ^b^Other mosquitoes tested: *Anopheles franciscanus*, *An. hermsi*, *Ae. vexans*, *Culiseta inornata*, *Cs. incidens*, *Cx. erythrothorax*, *Cx. erraticus*, *Cx. stigmatosoma*, *Oc. sierrensis*, *Oc. dorsalis*, *Oc. melanimon*, *Oc. taeniorhynchus*, *Psorophora columbiae*.

**Figure 4:**
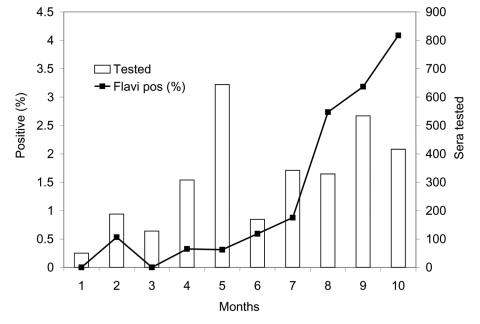
Wild bird *Flavivirus* seroprevalence rates (Flavi pos %) in Coachella Valley during 2003. Shown are percentages of total serum samples that tested positive each month by enzyme immunoassay. Positives include infections caused by West Nile virus and St. Louis encephalitis.

Serum samples from live free-ranging birds in Coachella Valley showed an increase in *Flavivirus* prevalence (Figure 4) in resident species (Table 2), with WNV, SLEV, and WEEV detected near sites where these viruses were isolated from mosquitoes or detected by sentinel chicken seroconversions (Figure 2). Confirmatory PRNTs showed that *Flavivirus*-positive birds were infected with both WNV and SLEV. Of 31 birds with demonstrable PRNT titers, 20 were infected with WNV, 8 were infected with SLEV, and 3 had equivocal titers against both viruses. Live bird sampling programs in Los Angeles, Bakersfield, and Sacramento did not collect antibody-positive birds despite comparable sampling and testing efforts (Table 1).

**Table 2:** Wild birds collected and bled in Coachella Valley, January 1–November 1, 2003

Species	Sera	% *Flavivirus*^a^	% WEEV^b^
Abert's Towhee	108	0.9	0.0
House Finch	251	0.4	0.0
Least Bittern	10	10.0	0.0
Gambel's Quail	643	3.3	0.2
Common Ground Dove	95	5.3	0.0
Mourning Dove	729	1.5	0.1
Domestic Pigeon	39	25.6	0.0
White-winged Dove	6	16.7	0.0
58 species	1,297	0.0	0.0
Total	3,178	1.6	0.1

^a^Positive by enzyme immunoassay (P/N ratio >2). Some EIA-positive sera were negative by plaque reduction neutralization test, whereas some others were postive, but there was <4-fold difference between West Nile virus and St. Louis encephalitis virus titers. ^b^WEEV, western equine encephalitis virus.

**Figure 5:**
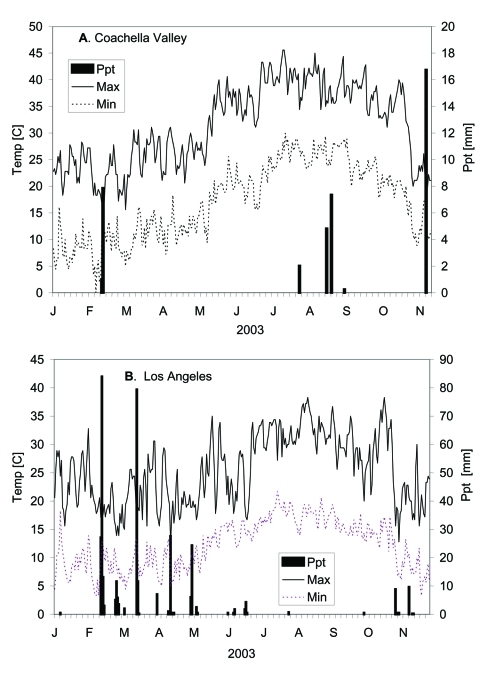
Climate conditions in A) Coachella Valley and B) Los Angeles at National Oceanic and Atmospheric Administration weather stations. Of interest was the dramatic drop in maximum temperature during early November coincident with the end of transmission. Ppt, precipitation.

Climatic conditions at the time of WNV introduction included above average temperatures and several rainfall events associated with the extension of the southwestern monsoon into southeastern California (Figure 5). Normally, summer storms track north from the Gulf of Mexico into Arizona and New Mexico; however, during the summer of 2003 a persistent high pressure system over Nevada resulted in a frequent clockwise pattern flowing from Colorado south into Arizona and then into southeastern California (http://www.srh.noaa.gov/abq/climate/Monthlyreports/July/nams.htm).

**Figure 6:**
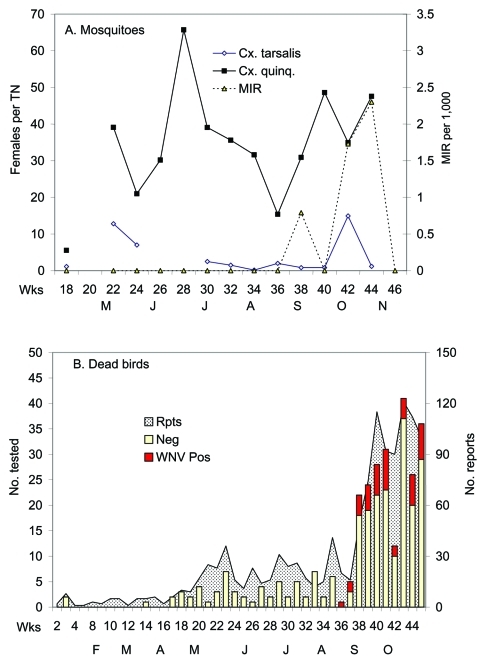
Virus temporal dynamics in relation to *Culex* abundance in the Whittier Narrows area of Los Angeles County. Shown are A) female *Cx. tarsalis* collected per CO_2_ trap night (TN) and female *Cx. p. quinquefasciatus* collected per gravid TN, West Nile virus (WNV) minimum infection rates (MIR) per 1,000 *Cx. p. quinquefasciatus* tested, adjusted for differential sample sizes, and B) number of dead birds reported, tested, and positive for WNV in Los Angeles County.

WNV then dispersed from the Salton Sea area to the City of Riverside in Riverside County and to the City of Arcadia in the Los Angeles Basin during September and October (Figure 2). In urban Los Angeles, WNV was tracked by testing dead birds reported by the public and by virus isolations from *Cx. pipiens quinquefasciatus* collected by gravid female traps (Figure 6). Sentinel chickens situated near dead bird collection sites remained negative for WNV, although two chickens in Monterey Park, Los Angeles County, seroconverted to SLEV during the week of September 16, 2003. Virus movement into the City of Riverside was associated with the detection of the first locally acquired WNV human case in California, followed by single cases in Imperial County and then the City of Whittier in Los Angeles County.

WNV then seemed to disperse south and was tracked through dead birds submitted from inland suburban communities along Highways 215 and 15 from San Bernardino to San Diego (Figure 2). Included in the 57 dead birds that tested positive for WNV through October 30, 2003, were 47 American Crows, l Brewer's Blackbird, 2 House Finches; 3 House Sparrows, l Northern Mockingbird, l Western Scrub-Jay, and l White-crowned Sparrow. WNV-positive dead raptors have yet to be reported, and sick or dead birds have not been reported from the Los Angeles or San Diego Zoos.

## Discussion

Enzootic monitoring by the California Encephalitis Virus Surveillance Program and associated field research projects provided an effective early warning that detected the introduction of WNV into rural southeastern California before reported avian, equine, or human illness. Our observations provided information related to the potential modes of dispersal and amplification as well as the effectiveness of different surveillance indicators to track WNV.

### Dispersal

The timing of initial WNV detection in California provided some insight into possible mechanisms for invasion and subsequent dispersal. WNV was first detected during mid-July in southeastern California concurrent with the detection and amplification of endemic SLEV. These events occurred approximately 7 months after the termination of reproductive diapause (22) and 2 months after the vernal peak in the *Cx. tarsalis* population (11), 2 months after the end of the nesting season for most resident avian species (18), 2 months after the passing of the northbound avian migrants, and 2 months before the arrival of the southbound avian migrants. This pattern of arbovirus appearance during midsummer, when temperatures are highest and vector populations lowest, has been documented repeatedly for SLEV in southeastern California and frequently occurs concurrent with the onset of the hot summer period associated with the southwest monsoon (12). Partial sequencing of SLEV isolates from southeastern California has indicated minimal genetic change during sequential years with SLEV activity but differences from isolates made after years with no virus detection (12,23) and from strains sequenced from Central and South America (24). Recently, minor genetic change has been detected in WNV isolated in the Yucatán (3).

Our attempts to detect WNV infection in both north- and southbound migrants along the Pacific flyway were unsuccessful, agreeing with our previous studies with SLEV and WEEV (18). Surveillance along the Pacific flyway from British Columbia Province, Canada, the northwestern United States, and western states in Mexico indicated that there was no WNV activity in these areas during the fall of 2002 or the spring of 2003 to provide a source of infection for migratory birds. In contrast, seropositive resident and migratory birds have been documented along the Atlantic and Mississippi flyways into the Caribbean (5) and tropical eastern Mexico (3,25), indicating WNV dispersal into these areas. During 2003, a total of 4,502 free-ranging birds from Sacramento, Kern, and Los Angeles Counties were tested for WNV antibody with negative results. An additional 3,178 birds collected in the Coachella Valley were tested through November 2003; 51 resident species had antibody to flaviviruses detected by EIA. Mourning Doves repeatedly were positive, and, although adults were present in Coachella Valley year-round, evidence from U.S. Geological Survey band recovery reports indicated considerable dispersal (23). Adult doves survive WNV infection and produce a moderate 3–5 log_10_ PFU/mL viremia of 5 days' duration (26) (W.K. Reisen, unpub. data).

The late summer increase in WNV transmission and dispersal coincided with postnesting movements by summer and year-round resident birds. Several passerine species, such as House Finches, form flocks at this time that forage widely and roost in various locations. Vagrants from these populations could be responsible for the movement of virus in rural agricultural sites. During the hot summer months, a short extrinsic incubation period in local vector populations feeding on sick and less mobile individual birds from these flocks could infect other local birds, resulting in the relatively rapid movement of virus by resident avian species.

Climate patterns can influence mosquito dispersal. Storm fronts previously have been proposed as dispersal mechanisms for mosquitoes and the arboviruses they transmit in Asia (27) and North America (28,29). Each summer, the southwest monsoon brings moisture from the Gulf of Mexico into the arid Southwest, and this movement often is characterized by intense local thunderstorm activity. High barometric pressure established over Nevada during 2003 created a persistent clockwise airflow pattern from Colorado into southeastern California through Arizona and northern Mexico. Surveillance in Arizona during 2003 detected WNV concurrent with that in southeastern California, perhaps indicating that a similar climate-driven mechanism brought virus southwest from the Colorado epicenter.

**Figure 7:**
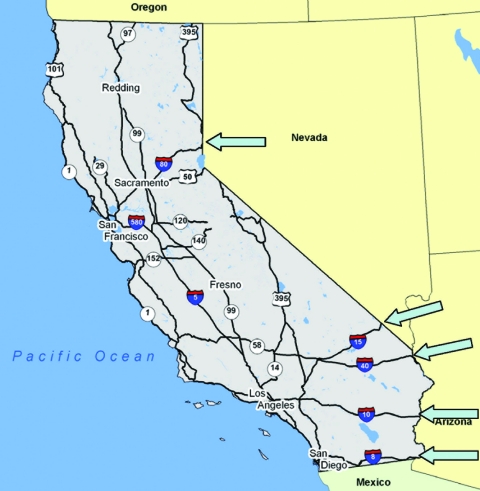
Road map of California. Arrows indicate the points of entry of main U.S. highways into California from the East.

A final and perhaps more remote consideration in the East-West dispersal of WNV is the transport of infected mosquitoes by commerce. The main East-West highways in the United States, such as I-15, I-40, I-10, and I-8, enter southern California (Figure 7). Possibly produce or other trucks loading at night or early morning in areas of intense transmission could entrap infected mosquitoes that would disembark when truck contents are inspected or off-loaded. If conditions for mosquito survival were suitable, these infected mosquitoes could be the source of virus introduction into new areas. Such a mechanism was considered among several possibilities as the source of several new mosquito species introductions into southeastern California (30,31). In this context, it is possible to conceptualize the introduction of WNV into southern California via I-8, followed by movement northward along Highway 86 into refuges near the Salton Sea in Imperial and Coachella Valleys, and then along I-10 and Highway 60 into Los Angeles and Riverside, respectively, and by movement down I-15 into San Diego. However, the WNV epicenter during 2003 was situated in the Colorado-Nebraska area, and most ground transport from this area would be expected to enter California by I-80 into the Sacramento area, where WNV has yet to be detected.

### Amplification

Three foci of virus amplification were studied (Figure 2). Based on our surveillance data, WNV amplification in rural southeastern California initially occurred throughout Imperial Valley and around the northern shore of the Salton Sea in Coachella Valley. Based on virus isolations, *Cx. tarsalis* was the vector species and resident birds the presumed amplifying hosts in this rural irrigated desert biome. Recovery of WNV from *Cx. tarsalis* was expected because this species was infected frequently with SLEV and WEEV during previous ecologic studies (11,12,32) and ongoing surveillance in rural southeastern California. Although susceptible to infection (33), other species, including *Cx. p. quinquefasciatus*, *Cx. erythrothorax*, and *Ae. vexans* collected concurrently were not infected with WNV. Avian serosurveys showed highest antibody prevalence rates among resident columbiform and galliform species, which produce moderate-to-low viremias and do not die from infection (26). The lack of passerine positives may reflect elevated death rates among these species; however, few dead birds were reported from these areas, and none tested positive for WNV. The limited number of corvid species and the sparse human population in this desert environment may have combined to limit the utility of dead bird surveillance.

Once WNV dispersed into urban Los Angeles, virus was isolated from dead birds reported by the public and from *Cx. p. quinquefasciatus* collected by gravid traps. Positive bird species included mostly American Crows as well as small-sized species such as House Finches and House Sparrows. The Whittier Narrows and associated riparian corridors appeared to be the site of WNV introduction and subsequent amplification. This area supports a large American Crow communal roost during the postnesting season in late summer and fall that may have contributed to the receptivity of this area for WNV introduction and subsequent amplification.

### Surveillance

WNV was monitored by using a wide variety of methods that varied in effectiveness. In rural southeastern California, WNV was tracked best by testing pools of *Cx. tarsalis* collected by CO_2_ traps and by monitoring sentinel chicken sera. Free-ranging birds, such as quail and doves, which do not succumb to infection, also were useful sentinels; however, differentiating WNV from SLEV infections was problematic for birds collected before a definitive rise in immunoglobulin G antibody titer. None of these surveillance methods worked well in urban or periurban areas of Los Angeles. Few mosquitoes, including *Cx. tarsalis*, were collected there by CO_2_ traps, and most positive pools to date have come from female *Cx. p. quinquefasciatus* collected by gravid traps. In urban neighborhoods, CO_2_ traps and other methods collect relatively few mosquitoes in comparison to gravid traps (34,35).

The dense human population in Los Angeles County reported >1,200 dead birds by the end of October; 218 of these were tested, and 38 were positive for WNV. As expected because of their susceptibility and large size, most positives were crows, but small-sized passerines also tested positive. In urban Los Angeles, sentinel chickens did not seroconvert to WNV during 2003, despite being situated near recoveries of WNV-positive dead crows and *Cx. p. quinquefasciatus* pools and being in the vicinity of the large Whittier crow roost. Differences in sentinel chicken sensitivity between rural and urban habitats may relate to vector mosquito dispersal and not to avidity for feeding on chickens. In agreement, of 78 serum specimens taken from backyard chickens of unknown age from this urban area along the Rio Hondo and San Gabriel riparian corridors, 7 had antibody confirmed by PRNT to be WNV. In California, *Cx. tarsalis* is very dispersive (36,37) and hunts along riparian corridors or vegetative transitions (38,39), whereas *Cx. p. quinquefasciatus* is less dispersive in urban environments and remains near the point of emergence (40). Therefore, infectious *Cx. p. quinquefasciatus* may be less likely to disperse in urban environments and encounter confined sentinel flocks than are *Cx. tarsalis* in rural environments, where farmhouse environs provide widely spaced "islands" of elevated vegetation used by birds for roosting and nesting and by *Cx. tarsalis* for host-seeking and resting. Southern California environments lack the contiguous canopy found in the eastern deciduous forest, and *Culex* mosquitoes feed readily at ground level (41,42). Therefore, positioning sentinels at ground level does not appear to have been a critical factor in effectiveness.

The number of dead bird reports in Los Angeles increased after WNV was introduced, presumably because of media coverage, public education concerning the dead bird surveillance program, and increased WNV-associated bird deaths. Our laboratory data indicated that approximately 80% of the dead birds tested after the invasion and media publicity were WNV-negative. These data indicated that at low-to-moderate levels of enzootic transmission, dead bird reports alone may not be a true indication of the level and location of WNV transmission. In addition, use of antibody testing of free-ranging birds collected in grain-baited crow traps (mostly House Sparrows and House Finches) did not seem to be a productive surveillance method in Los Angeles, and all birds to date have tested negative, including those trapped at Whittier Narrows.

Our data during 2003 clearly showed that WNV introduction, amplification, and widespread dispersal occurred with few human or horse cases, indicating that such cases are insensitive indicators of WNV presence and enzootic activity levels. Most humans in rural southern California reside in homes with some form of air-conditioning and thereby may be protected from mosquito contact during the evening (43). Unknown proportions of horses in California are vaccinated and thereby may be protected from disease. Epidemic transmission of WNV in southern California has been predicted for 2004, and it will be of interest to determine how well enzootic measures of virus activity forecast human infection.

### Response

California health agencies and vector control districts have been preparing for the introduction of WNV since movement into the West seemed eminent, and state guidelines for escalated control responses to surveillance data have been prepared (http://westnile.ca.gov/Publications.htm). Initial responses included enhanced surveillance, expanded larval control operations, and preparation for emergency adult control. Extended surveillance in Imperial County by the Imperial County Health Department, Coachella Valley Mosquito and Vector Control District, and University of California, Davis, and the development of a dead bird surveillance program by the California Department of Health Services during 2002 are examples of new programs that proved useful in tracking WNV during 2003. Detection of WNV in southeastern California during 2003 triggered adult mosquito control operations to interrupt transmission at wetlands and to protect residents of the small towns of Niland in Imperial County and Mecca in Coachella Valley. Dead bird surveillance data in urban Los Angeles were used to direct focal larval control operations and to launch public education programs through various media events. Surveillance activities in southern California continued during the winter of 2003 to 2004 and have included mosquito pool submission, sentinel chicken testing, live bird sampling and testing, and dead bird reporting and testing. All findings have been negative through mid-February 2004, despite surveillance near wetlands along the Salton Sea and at the Whittier Narrows crow roost, perhaps indicating that transmission ceased, despite mild winter conditions. Positive after-hatching-year and second-year resident birds from Coachella Valley have been collected, but these birds presumably were infected during 2003; all winter resident birds, such as White-crowned Sparrows, have remained negative. Planned and ongoing operational responses during spring 2004 have been coordinated at the local, regional, and state levels but necessarily vary among agencies because of local ecology, politics, and funding. The introduction of WNV into California and its anticipated amplification during the next few years will provide a rigorous test of how well a widespread integrated vector management approach to mosquito control can protect the residents of California from mosquito-borne disease.
